# Editorial: Deep Brain Stimulation Think Tank: Updates in Neurotechnology and Neuromodulation, Volume II

**DOI:** 10.3389/fnhum.2022.912730

**Published:** 2022-06-01

**Authors:** Adolfo Ramirez-Zamora, James Giordano, Casey Halpern, Christopher Butson, Michael S. Okun

**Affiliations:** ^1^Norman Fixel Institute for Neurological Diseases, University of Florida, Gainesville, FL, United States; ^2^Departments of Neurology and Biochemistry, Neuroethics Studies Program—Pellegrino Center for Clinical Bioethics, Georgetown University Medical Center, Washington, DC, United States; ^3^Department of Neurological Surgery, Perelman School of Medicine at the University of Pennsylvania Richards Medical Research Laboratories, Philadelphia, PA, United States

**Keywords:** deep brain stimulation, Parkinson's disease, adaptive neuromodulation, neuroethical considerations, depression, neuroimaging, magnetic resonance image

## Introduction

The introduction of ever-newer technologies, improved software, and an increasing understanding of the cerebral anatomic and physiologic substrates involved in neurological and psychiatric conditions have all advanced research in neuromodulation, and its viable translation to clinical practice. In an effort to create a forum and nexus for stake and shareholders in techniques and technologies of deep brain stimulation (DBS) the group evolved into a freely interacting multidisciplinary group assembled to discuss challenges, problems, progress, and opportunities in the field. The first DBS Think Tank was convened in 2012 at the University of Florida, Gainesville FL. Since that initial meeting, the DBS Think Tank has grown, through the hybrid use of virtual and in-person resources to expand the involved number, and scope of worldwide participants from research, engineering, clinical, ethical-legal, and commercial disciplines. Since 2013, proceedings of the DBS Think Tank have been published and these highlight the most current and emerging work in the field. These published proceedings are open access and available to the public (https://fixel.ufhealth.org/research/deep-brain-stimulation-think-tank/think-tank-published-proceedings). Recognizing that different geographical regions often face unique needs and challenges, and to better understand the specific opportunities and limitations of DBS approaches upon the contemporary global stage, several researchers from Asia and Oceania initiated a separate meeting mirroring the spirit and structure of the original DBS Think Tank. The first East DBS Think Tank took place in June 2019 in Kyoto Japan, and this was followed by a virtual meeting in China in December 2020 (due to travel constraints imposed by the COVID-19 pandemic).

The DBS Think Tank should be seen as a genuine effort to conjoin multi-disciplinary perspectives to collaboratively crowd views, values, and issues in DBS research. Presentations and discussions have addressed a range of topics, including advocacy for DBS; improving clinical outcomes; technical and methodological, innovations and advancements; broadened understanding of neurophysiology, and neuropathology; and ethical questions, problems, and their potential solutions. As open dissemination of developments in DBS is both needed and critical for the advancement of science as a viable social good. Ongoing collaboration with Frontiers Editorial Office has afforded rapid yet nonetheless detailed review of work in the field.

In this spirit, this Editorial focuses upon the second volume. Twenty-three manuscripts were accepted within four different categories: (1) Clinical outcomes and DBS practice, (2) Neuromodulation for neuropsychiatric conditions [with particular emphases upon depression and OCD], (3) New insights toward integrating neuroimaging and DBS and (4) Progress in incorporating other neurotechnology in DBS research and clinical applications.

The Eighth annual DBS Think Tank Proceedings have been published. Vedam-Mai summarizes the discussions that took place in September 1st and 2nd, 2020. As in previous years, the meeting reviewed currently available advances in commercially offered DBS devices, and dedicated a section to discussing the ethical implications of (1) using DBS for rare diseases; (2) providing continued access to DBS—and supportive neuroscientific and technological methods after trials are completed; ongoing and future activities of the NIH BRAIN Initiative (inclusive of those NIH enterprises in ethics that are focused upon DBS). Discussions of the status of DBS for management of depression, development and use of novel approaches to identifying neurological node and network dysfunction in depressive signs and symptoms, and the use of neurophysiologic and neuroimaging techniques and tools to refine DBS targeting (see also below). Other advances were addressed and included the use of precision imaging and connectomic surgery; adaptive DBS; optogenetics methods for facilitating improved understanding of the molecular neurobiology of diseases; and the use of local field potentials (LFPs) as biomarkers for DBS control and programming.

## Advances in DBS Clinical Practice

Zhang et al. from Ruijin Hospital, Shanghai Jiao Tong University, Shanghai, China share their patients' experiences of the challenges encountered with DBS treatment during the early stages of the COVID pandemic. Indubitably, cancellations and delays of DBS surgeries were common as part of the initiatives to prevent the spread of the virus, and this disrupted the provision of neurological care—a reality that occurred not only in China, but subsequently in many other parts of the world. Of note was that that patients seeking DBS surgery during the initial phase(s) of the pandemic were predominantly as consequence of routine clinical referral; personal safety that could be provided by hospital care; and poor control of severe neurological symptoms through the use of other therapeutic modalities.

Multiple authors from different institutions, and presented by Mahajan et al. provide results of a comprehensive 58-question web-based practitioner survey conducted between December 2015 and May 2016 that focused upon DBS referral practices and peri-operative management. These results reveal considerable variability in the perceived best approaches for DBS selection, target selection, procedure type, and postoperative practices. As well, small, but significant differences in practice were noted across global regions, with differential utilization of multidisciplinary teams, and various (mood and cognitive) assessments prior to surgery.

Molina et al. report their experience using closed-loop DBS to treat medication-refractory freezing of gait (FoG) in Parkinson's disease (PD). Management of FoG. a paroxysmal phenomenon that provides an ideal framework for the possibility of “on demand “ closed-loop DBS (CL-DBS), was noted to be challenging, with limited benefit achieved by accessing current targets [viz.- the pedunculopontine nucleus (PPN) for medication-refractory FoG]. Molina et al. compared the preoperative number of FoG episodes vs. the number of FoG episodes at 6 months post-DBS at the optimized settings in a gait lab. While the primary outcome variable was met in three of the five subjects who exhibited a >40% improvement in the number of FoG episodes from baseline to 6 months when on acute PPN CL-DBS, there were no significant differences between the pre-DBS and month 6 FoG counts at the group level. Moreover, there were numerous reports of side effects in this cohort, with 40% explantation due to delayed infection.

Investigators at the Cleveland Clinic and Case Western University in Ohio examined changes in PD patients' desired level of control of their DBS, and perception of global life control throughout DBS (Merner et al.). Participants reported decreased desired control of stimulation throughout DBS treatment, and significantly greater global life control. These findings highlight important distinctions between particular aspects of control, and suggest that patients may be more willing to share or cede certain domains of control as they gain greater global life control consequential to DBS intervention.

Sarica et al. provide a comprehensive review of key hardware and software specifications of commercially available IPG systems; offering a detailed account of challenges and developments related to DBS hardware, and highlighted strategies to improve IPG longevity and other practical problems.

Wong et al. detail the use of burst-cycling deep brain stimulation (BCDBS) for the management of FOG in PD. They reported benefit of BCDBS that was comparable to conventional DBS in measures of FOG, gait, functional mobility and other motor symptoms. These results support BCDBS as a feasible, safe, and well-tolerated intervention with considerable potential as a viable future DBS programming strategy.

The neuromodulation group at the University of Florida studied the effect of DBS on pallidal oscillatory activity and symptom severity in a PD patient implanted with the Medtronic Percept system (Cagle et al.). Using recordings of pallidal LFPs while delivering stimulation in a monopolar configuration using stepwise increments (0.5 mA, every 20 s), it was found that electrical stimulation delivered to the target region elicited beta desynchronization. Beta power was strongly correlated to improved bradykinesia (when measured in the acute clinic setting). Interestingly, it was noted that beta power *rebounded* when the stimulation amplitude was increased, and this was associated with worsening bradykinesia. Although the mechanism for this phenomenon is unknown, their results can provide useful information to parametrize therapeutic windows for DBS programming.

Adult-onset truncal dystonia (ATD) is a rare presentation of this disorder, accounting for ~10% of segmental dystonia affecting the trunk, inclusive of the paraspinal and abdominal wall muscles. ATD presents a clinical challenge, as response to treatment has been limited to date. Few reports have specifically addressed the potential role of DBS in the management of dystonic opisthotonos in the context of truncal predominant adult-onset dystonia. In this light, Tambirajoo et al. present outcomes of (three patients with) ATD managed with pallidal DBS, who showed a rapid and sustained clinical improvement of their symptoms with postoperative follow-up of 2–3 years.

Chen et al. discussed the importance, role, and value of large-scale data infrastructure in developing next-generation DBS therapeutics. Increasing challenges of managing massive (multi-scalar, and diverse) data include issues and problems in data acquisition, storage, organization, analysis, which are each and all instrumental to integrating complex neural time-series data with dynamic assessments of patients' clinical signs and symptoms. The authors reviewed Rune Lab, a scalable, HIPAA-compliant, cloud-based data platform designed for (1) time-synchronization and aggregation of multi-modal datasets (2) real-time data access, and (3) data analysis at the multiple terabytes scale directly in the cloud; and concluded that the system architecture, development process, and viability of shared data platforms afford considerable utility and value in both DBS research and clinical utility.

## DBS Therapeutics for Neuropsychiatric Conditions

Major depressive disorder is a common, often disabling disorder with high rates of treatment resistance, for which DBS continues to be explored as a valuable potential intervention. There is increasing evidence that depression is characterized by distributed network dysfunction that extends beyond a single brain region or neurochemical system. Computational advancements employing a network neuroscience framework have enabled brain activity to be modeled with greater granularity and complexity so as to better understand such distributed processes.

Scangos et al. studied whether application of a novel computational approach to large sample, high spatiotemporal resolution neural recordings in humans could demonstrate the functional organization and coordinated activity patterns of neurological networks involved in clinical depression. Using intracranial mapping with multi-channel iEEG for seizure localization as part of standard medical care while collecting clinical data regarding depressive symptoms, they elucidated two putatively contributory subnetworks. The first was characterized by left temporal lobe hypoconnectivity and pathological beta activity; the second was characterized by a hypoactive, but hyperconnected left frontal cortex. These novel findings have important implications for diagnosis, subtyping, and planning and monitoring treatment of depressive disorder(s).

Thomson et al. provided an exploratory study that employed a prospective qualitative design, and iterative thematic analysis to assess both patient perspectives of, and goals for DBS treatment (targeting the bed nucleus of the stria terminalis) of depression. It was found that patients' decision to undergo DBS was characteristically motivated by the intolerability of life with severe depression, and the exhaustion—and ineffectiveness—of other available treatment options. It was also reported that many patients expressed surprised by the lengthy process of establishing optimum stimulation settings, and felt the intervention was a “work in progress.”

## New Insights to the Combined Use of Neuroimaging and DBS

Schrock et al. presented a case report that reviewed the importance of lead localization within the targeted nucleus for achieving effective clinical benefit. Using 7T MRI and computational modeling it was shown that severe mood-related side effects (with minimal motor improvement) occurred in a PD patient following DBS in the limbic/associative territory of the STN. The patient experienced marked improvement in motor benefit, and resolution of mood side effects following repositioning of the lead within the STN sensorimotor territory. These findings served as a basis for a patient-specific anatomical model (provided in outstanding graphic depiction) of the STN with parcellation into distinct functional territories, which enabled computational modeling to evaluate the extent and effect(s) of activating particular target sites.

Chang et al. present their data from a single subject, and discussing the potential use of DBS of a closely related nucleus dorsal to the PPN—the cuneiform nucleus (CnF) as potentially important for gait control. Targeting guided by diffusion tensor imaging (DTI) and anatomical landmarks afforded neurosurgical details for targeting, which produced improved outcome metrics in gait, and in short-term reduction of FOG, which certainly warrant additional follow up studies.

Morishita et al. provide a case report of a patient with facial and palatal tremor due to craniofacial dystonia, and use normative connectome analysis to determine activation of specific fiber tracts *via* pallidal vs. thalamic DBS. Their results revealed that the fiber tracts associated with VTA of GPi DBS had different connections with the facial area of the motor cortex, which could explain differences in clinical outcomes, and help to guide future DBS intervention(s).

## Progress in Incorporating Neurotechnology in DBS

Tabacof et al. report the safety of a wearable, vibrotactile stimulation device for treating tremor in PD, noting that treatment of resting tremor is ineffective in a significant number of patients. In this work, a vibrotactile stimulation was delivered bilaterally to the wrists and ankles using four custom-built, wearable devices to evoke optimal full body vibrotactile stimulation. This system was shown to produce a moderate effect on tremor, with no reported adverse events, and appears to be safe and well-tolerated.

The effect of DBS on cerebellar vs. non-cerebellar tremor in a patient with multiple sclerosis was discussed by Xie et al. In this case report, a wearable accelerometer was applied to the index finger of each hand to quantitatively characterize kinetic tremor frequency and amplitude at the initiation and cessation of hand movement in a patient treated with thalamic DBS. In comparing both limbs in the ON and OFF stimulation state, they noted good responses, with reduction of cerebellar tremor, but only limited effect—with minimal functional benefit—on distal limb oscillation.

Chronically implanted, bidirectional, neural interfaces provide unprecedented access to, and assessment of human neurological function during activities of daily living in a range of disease and symptom states. To successfully optimize therapy for patients implanted with these devices, analyses must be conducted offline of the recorded neural data. The format, volume, and complexity of raw data from these devices necessitate conversion, parsing, and temporal reconstruction in advance of the time-frequency analyses and modeling required for evaluation toward such ends, Sellers et al. provide an open-source MATLAB toolbox capable of taking raw files (from the Summit RC+S device, available under investigational device exemption and employed in a range of clinical indications), transforming the data, and providing salient outputs and user functionality. This could be important for both researchers and clinicians, particularly as new commercial devices allow for prolonged (ecologically valid), assessment of brain signals relevant to sustainable therapeutic outcomes.

Taken together, these contributions afford a view of a leading edge of DBS research and its translational applications in clinical care. As mentioned above, a patient astutely noted that DBS is indeed a “work in progress.” To be sure, the field and approaches gain precision and momentum from the cooperative efforts of the groups of engineers, scientists, clinicians, and those who inform and develop guidelines and policy to support ongoing experimentation, and therapeutic improvement. As we approach the decadal anniversary of launch of the US BRAIN Initiative (available online at https://www.braininitiative.org and https://obamawhitehouse.archives.gov/BRAIN) we believe it is important to let its titular invocation of “advancing innovative neurotechnology” serve as the cornerstone for investigation, invention, and safe, ethically sound clinical intervention. It is our hope that the DBS Think Tank—along with other focally dedicated efforts—will continue to provide nexus and vectors for such progress.

## Author Contributions

AR-Z, JG, CH, CB, and MO: draft manuscript preparation and revisions. All authors reviewed the results and approved the final version of the manuscript.

## Conflict of Interest

The authors declare that the research was conducted in the absence of any commercial or financial relationships that could be construed as a potential conflict of interest.

## Publisher's Note

All claims expressed in this article are solely those of the authors and do not necessarily represent those of their affiliated organizations, or those of the publisher, the editors and the reviewers. Any product that may be evaluated in this article, or claim that may be made by its manufacturer, is not guaranteed or endorsed by the publisher.

